# Maternal postpartum six‐week check and short‐term health outcomes for women with hypertensive disorders in pregnancy: An observational study using the Clinical Practice Research Datalink (CPRD)

**DOI:** 10.1111/aogs.15068

**Published:** 2025-02-25

**Authors:** Rema Ramakrishnan, Diane Korb, Yangmei Li, Marian Knight, Claire Carson

**Affiliations:** ^1^ National Perinatal Epidemiology Unit, Nuffield Department of Population Health University of Oxford Oxford UK; ^2^ CRESS, Obstetrical Perinatal and Pediatric Epidemiology Research Team Université de Paris, EPOPé, INSERM, INRAE Paris France; ^3^ Obstetrics and Gynecology Department, Hôpital Robert Debré, Public Assistance‐Paris Hospitals Université Paris Cité Paris France; ^4^ NIHR Policy Research Unit in Maternal and Neonatal Health and Care, National Perinatal Epidemiology Unit, Nuffield Department of Population Health University of Oxford Oxford UK

**Keywords:** health services, hypertensive disorders of pregnancy, maternal health, postpartum care, primary care

## Abstract

**Introduction:**

In the UK, in addition to the recommended blood pressure check, women with hypertensive disorders of pregnancy are offered a postpartum six‐week check (SWC) with either a specialist or general practitioner. We aimed to describe the prevalence and disparities in the provision of SWC, and describe short‐term postpartum outcomes among women with hypertensive disorders of pregnancy by SWC status.

**Material and Methods:**

Data were from the UK Clinical Practice Research Datalink GOLD for women aged 15–49 years at childbirth in 2000–2018, who were diagnosed with hypertensive disorders of pregnancy, and were registered for ≥12 months postpartum. Trends in SWC prevalence and differences in characteristics and short‐term postpartum outcomes of women by SWC status were described. Multivariable modified Poisson regression was used to compute risk ratio of a SWC for severe hypertensive disorders of pregnancy.

**Results:**

Of 30 483 women, 61.4% had a SWC. The proportion of women who received a SWC check was highest in 2007 (66.8%) but subsequently decreased. Women were less likely to have a SWC record if they were younger, of a minoritised ethnicity, living in more deprived areas, were multiparous, had severe hypertensive disorders of pregnancy, preterm birth, caesarean birth, or had a low birthweight baby. Compared to women who did not have a SWC, a higher proportion of women with SWC had their blood pressure recorded (SWC 47.4%, no SWC 39.9%), and had a diagnosis of postpartum depression (SWC 13.6%, no SWC 11.1%). There were no substantial differences in other short‐term postpartum outcomes by SWC status.

**Conclusions:**

There may be missed opportunities in postpartum care among women with hypertensive disorders of pregnancy. Our findings highlight the need to ensure that general postpartum care is not overlooked in women with specific morbidities in pregnancy.

## Graphical abstract

In this higher‐risk population of women with hypertensive disorders of pregnancy, disparities exist in the provision or uptake of six‐week postnatal checks. Women without a six‐week check were younger, from deprived areas, have severe disease, or a difficult birth, suggesting missed opportunities in postpartum care.

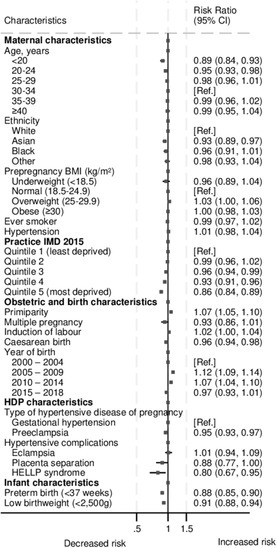

**Abbreviations**
BP: blood pressure
CI: confidence interval
CPRD: Clinical Practice Research Datalink
CVD: cardiovascular disease
EHR: electronic health record
GP: general practitioner
HDP: hypertensive disorders of pregnancy
RR: risk ratio

**Key message**

Disparities in six‐week postnatal checks among women with hypertensive disorders of pregnancy suggest missed opportunities in postpartum care for this higher‐risk population, emphasizing the need to address postpartum care for women with specific pregnancy‐related morbidities.

## 1 INTRODUCTION

With a prevalence of about 8%–10% in the UK, hypertension in pregnancy contributes to a significant burden of maternal and neonatal morbidity and mortality.^1^ Hypertensive disorders of pregnancy (HDP) include chronic hypertension, gestational hypertension, and preeclampsia. Measurements of systolic blood pressure (BP) ≥140 mm Hg and/or diastolic BP ≥90 mm Hg are considered hypertensive.^1^ Where women have readings in excess of 140/90 mm Hg prior to pregnancy, in the first 20 weeks of gestation, or are taking antihypertensive medication in this period, they are considered to have pre‐existing chronic hypertension.^1^ Gestational hypertension describes women who only develop hypertension after 20 weeks gestation. The presence of proteinuria or systemic involvement indicates preeclampsia, a more severe type of HDP, which can co‐occur with both chronic and gestational hypertension, and can be life‐threatening for both mother and baby.^1^

Pregnancy is often described as a physiological stress test, and HDP further increases the pressure on the heart and cardiovascular system. Along with short term implications of HDP for women and their babies such as mortality (maternal and neonatal), organ damage or failure, low birth weight, and stillbirth,^2^, ^3^ there is some evidence of longer term sequelae including an increased risk of persistent hypertension, major cardiovascular events such as myocardial infarction or stroke, and cardiovascular mortality.^4^

A health check for women at 6–8 weeks after the birth of a child has long been part of postpartum provision in primary care in England, although it has only been considered an essential service under the general practitioner (GP) contract since February 2020.^5^ All mothers, including those in higher risk groups, should be offered a six‐week check‐up (SWC) that covers various aspects of postpartum physical and mental health, and includes a BP check. However, the National Health Service guidance states that women with HDP should also be offered an appointment with a GP or specialist to check their BP at 6–8 weeks, which is separate to the SWC.^6^ Therefore, the focus of care for women with HDP will potentially be on their BP, and routine postpartum care may be overlooked as care for their specific conditions is prioritized. This may occur because clinicians focus on HDP, or because women attend for an HDP check and then do not return for a full postpartum check. It is therefore, perhaps plausible that women with severe HDP are not receiving general postpartum care.

This study explores whether women who have had HDP received their maternal postpartum check with a GP at 6–8 weeks and if this is associated with their health in the first year postpartum. The outcomes of interest include both short‐term outcomes related to HDP, and other postpartum health conditions that may be identified in a standard SWC.

The specific aims of this study were:

1. To describe the prevalence of a SWC with a GP among women with HDP, and examine the characteristics of women by SWC status.
2. To establish whether women who experience more severe HDP are more or less likely to receive their SWC after accounting for differences in age, area deprivation and ethnicity.
3. To describe short‐term outcomes related to HDP (subsequent BP monitoring and recording of abnormal BP in the 12 months postpartum; incident cardiovascular disease (CVD) in the 12 months postpartum; and differences in antihypertensive use) among women with HDP who do and do not have a SWC.
4. To describe common short‐term outcomes in the first year postpartum such as perineal or pelvic pain, urinary and fecal incontinence, sexual dysfunction, and postpartum depression among women who do and do not have a SWC.

## 2 MATERIAL AND METHODS

### 2.1 Data source

We used data from the UK Clinical Practice Research Datalink (CPRD) GOLD database linked to hospital episodes statistics, Office for National Statistics (ONS) linked deaths, and the Index for Multiple Deprivation data. The CPRD is an ongoing primary care database of patient‐level anonymised records from consenting general practices in the UK. These patients are representative of the UK population in age and sex^7^ and represented 6.9% of the total UK population in 2013. CPRD provides a pregnancy register identifying pregnancies using an algorithm based on codes for pregnancy‐related records.^8^

### 2.2 Study population

Women aged 15–49 years at childbirth were included in this study if they had a pregnancy identified in either the CPRD pregnancy register or linked hospital admitted patient care data, gave birth between Jan 1 2000 and Dec 31 2018, were diagnosed with HDP, had an active registration with a CPRD contributing GP practice considered of research quality at the time of childbirth and for 1 year postpartum, and had linked hospital episodes statistics data. In multiparous women, only the first pregnancy with HDP recorded was eligible. HDP was defined based on any one of the Read or International Classification of Diseases (ICD)‐10 codes listed in the study by Leon et al.^9^ These codes related to a diagnosis recorded in primary or secondary care of preeclampsia, gestational hypertension, superimposed preeclampsia, or pre‐existing hypertension during pregnancy after 20 weeks of gestation. We excluded pregnancies where the pregnancy register algorithm used postnatal records to define or adjust the date when pregnancy ended, since the SWC could not be accurately dated.

### 2.3 SWC definition

The definition of a SWC was identical to the one used in the paper by Li et al.^10^ where women were coded as having had a SWC if they had codes specific for SWC. In the absence of these codes, SWC was defined based on specified codes that indicated a postpartum check or physical examination in the 4–12 weeks postpartum.^10^

### 2.4 Outcomes

The outcomes examined included both cardiovascular‐related conditions (incident CVD during one‐year postpartum, BP status and antihypertensive medication following their SWC up until one‐year postpartum) and more general postnatal health (perineal or pelvic pain, urinary or fecal incontinence, sexual dysfunction, and postpartum depression during one‐year postpartum). Incident CVD was defined as the first diagnosis of CVD in CPRD and hospital episodes statistics during one‐year postpartum using previously validated and replicable CALIBER electronic health record code lists and algorithms (for details see https://www.caliberresearch. org/portal/). The definition of CVD included 12 diseases. These were: ischaemic stroke, intracerebral hemorrhage, subarachnoid hemorrhage, stroke not otherwise specified, myocardial infarction, stable angina, unstable angina, coronary heart disease not otherwise specified, peripheral arterial disease, abdominal aortic aneurysm, atrial fibrillation, and heart failure. For BP status, we created a 3‐category variable—normal, abnormal, or no BP measure. Abnormal BP was defined as having either a systolic BP ≥140 mm Hg or diastolic BP ≥90 mm Hg or both anytime following their SWC up until one‐year postpartum. A systolic BP value outside the range of 50 mm Hg–300 mm Hg was considered as an outlier and coded as missing.^11^ The Table S1 includes description of code lists used to derive variables for antihypertensive medication use, perineal or pelvic pain, urinary or fecal incontinence, sexual dysfunction, and postpartum depression.

### 2.5 Other variables

We included maternal characteristics such as age in years, ethnicity, pre‐pregnancy body mass index, smoking, chronic hypertension, patient‐level Index for Multiple Deprivation in quintiles; pregnancy and birth characteristics such as primiparity, multiple pregnancy, induction of labour, caesarean birth, and year of infant birth; details of HDP such as type of HDP (gestational hypertension and preeclampsia) and complications (pulmonary oedema, eclampsia, placenta separation, and HELLP syndrome); and infant characteristics such as gestational age at birth and birthweight. A woman who had hypertensive complications, or a caesarean birth or induction of labour before 34 weeks of gestation was considered to have had severe HDP.

### 2.6 Statistical analysis

The overall and annual prevalence of SWC was computed. Counts and percentages were used to describe the characteristics of women who had a SWC and did not have a SWC. Modified Poisson regression adjusted for confounders was used to identify whether women who experience more severe HDP are more or less likely to get their SWC. Previous analyses indicated age, ethnicity, Index for Multiple Deprivation and year of infant birth are all associated with the likelihood of having a SWC,^10^ and were considered as potential confounders. This regression analysis was conducted on women who had complete data for the covariates (*n* = 29 857; 587 missing for severity variable).

Outcomes (cardiovascular‐related conditions and general postnatal health) in women who had and did not have a SWC were described using counts and percentages. Additionally, for depression we examined the differences by timing of diagnosis during the one‐year postpartum to examine the potential for earlier detection in those who received a SWC. It was not possible to examine the differences by timing of diagnosis for other conditions because of small numbers.

Analyses were conducted using the statistical software, Stata version 18.

## 3 RESULTS

**FIGURE 1:**
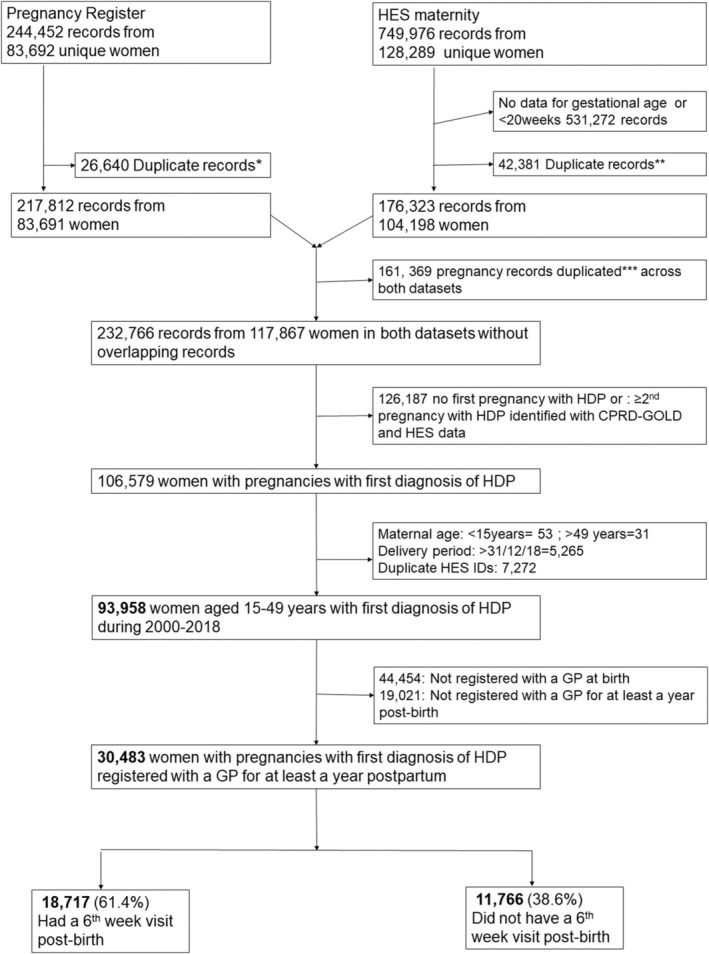
Flowchart of study participants.

**FIGURE 2:**
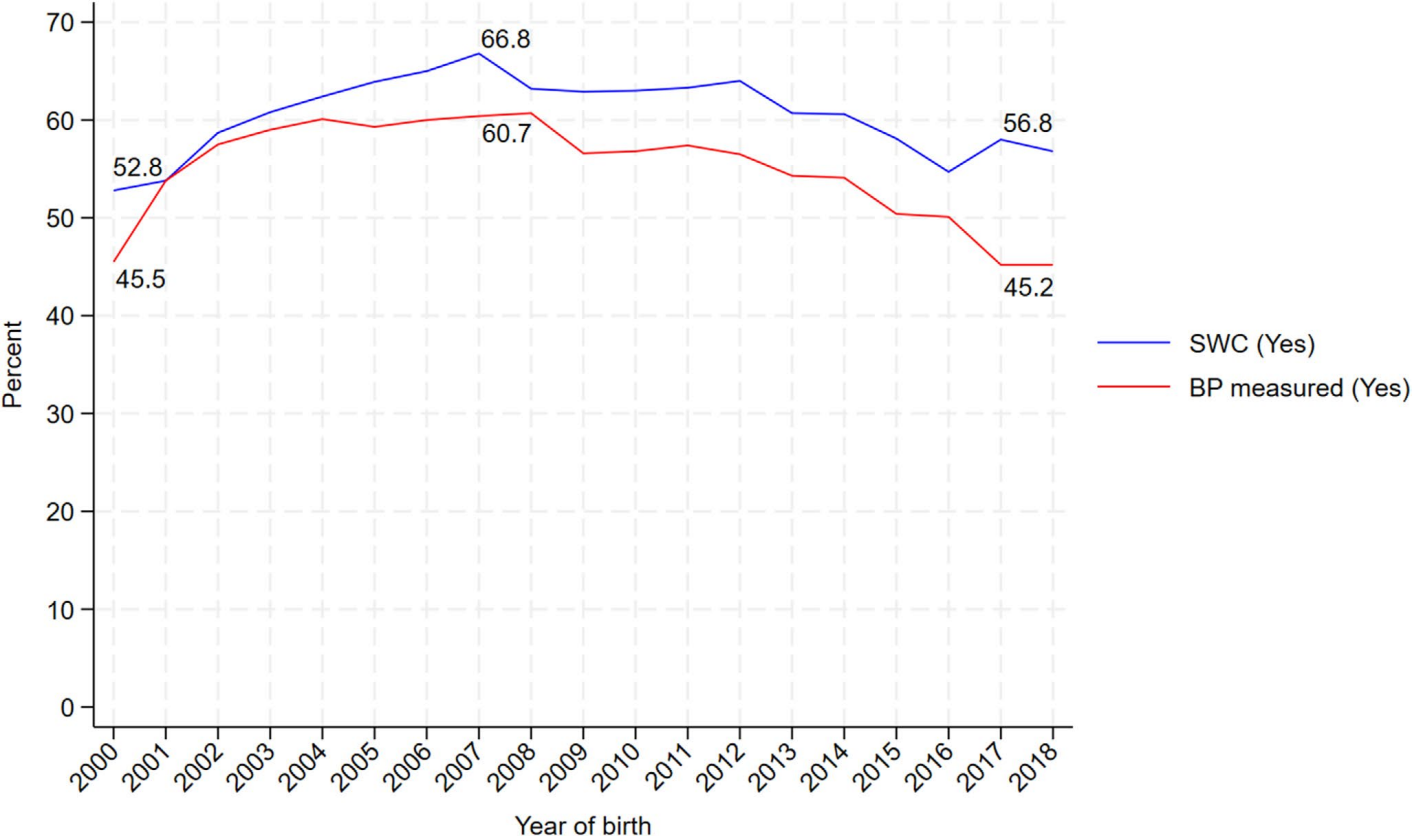
Trend over time in SWC visits and BP measured expressed as percentage.

Between 2000 and 2018, 93 958 eligible women who were registered in a CPRD‐contributing GP practice had evidence of a pregnancy with HDP recorded in either primary care or hospital admission records. During this period, there were 1 761 708 women in the source population; these figures resulted in an estimated prevalence of 5.3% for HDP. To improve the validity of the primary care data, we also required women to be registered at the CPRD practice at the time of the birth and for the subsequent 12 months postpartum (excluding 44 454 (47.3%) and further 19 021 (20.2%) pregnancies, respectively). The final analytic sample consisted of 30 483 women, of whom 61.4% had a record indicating a postpartum SWC (Figure 1 [flow chart]). Temporal plots showed that the prevalence of the SWC in women with HDP peaked at 66.8% in 2007, subsequently falling to 56.8% in 2018 (Figure 2).

### 3.1 Factors associated with not receiving a SWC

**FIGURE 3:**
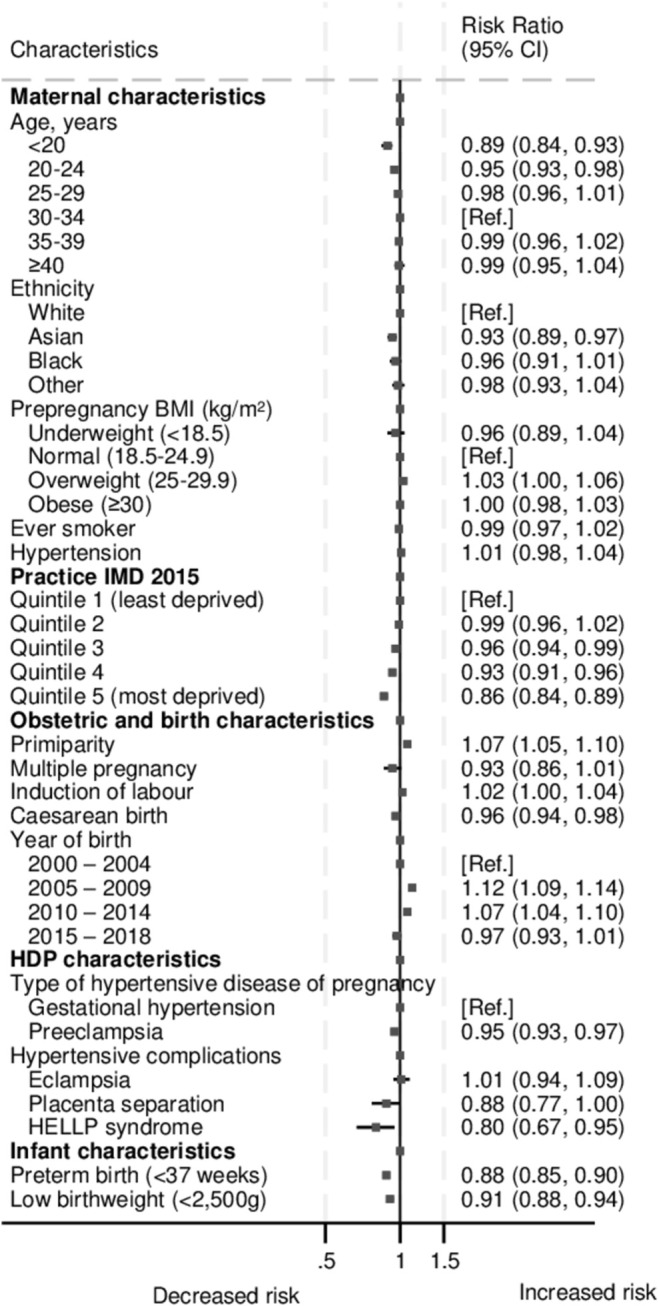
Crude risk ratio of six‐week check‐up (SWC) by maternal demographic and infant characteristics.

The prevalence of the SWC varied according to sociodemographic characteristics, with younger mothers, multiparous women and those living in more deprived setting less likely to have a SWC recorded (Table 1 and Figure 3). Preterm birth and caesarean birth were associated with a lower prevalence of SWC. Severity of HDP also appears to be a factor with women less likely to have SWC if they had severe disease (risk ratio (RR)_adj_ 0.86 (95% confidence interval [CI]: 0.82, 0.90)) (data not shown). Furthermore, SWC was less frequent in women who had preeclampsia, compared to gestational hypertension (RR_unadj_ 95%CI: 0.93, 0.97) (Figure 3). While absolute numbers of hypertensive complications such as eclampsia, placental separation or HELLP syndrome were small (Table 1), they were slightly more common in women who did not subsequently have a SWC (3.1% vs. 2.6%).

**TABLE 1:** Maternal demographic and infant characteristics by six‐week check‐up (SWC) status (*n* = 30 483).

	Did not have a SWC	Had a SWC	Total
	*n* = 11 766	*n* = 18 717	*N* = 30 483
	*n* (%)	*n* (%)	*n* (%)
Maternal characteristics			
Age, years			
<20	831 (7.1)	963 (5.2)	1794 (5.9)
20–24	2065 (17.6)	2994 (16.0)	5059 (16.6)
25–29	3086 (26.2)	4817 (25.7)	7903 (25.9)
30–34	3350 (28.5)	5757 (30.8)	9107 (29.9)
35–39	1904 (16.2)	3280 (17.5)	5184 (17.0)
≥40	530 (4.5)	906 (4.8)	1436 (4.7)
Ethnicity			
White	10 068 (87.6)	16 183 (88.7)	26 251 (88.3)
Asian	635 (5.5)	876 (4.8)	1511 (5.1)
Black	446 (3.9)	640 (3.5)	1086 (3.6)
Other	341 (3.0)	556 (3.0)	897 (3.0)
Missing (%)	2.4	2.5	2.4
Prepregnancy BMI, kg/m^2^			
Underweight (<18.5)	201 (3.0)	302 (2.4)	503 (2.6)
Normal (18.5–24.9)	2746 (41.2)	5012 (40.3)	7758 (40.6)
Overweight (25–29.9)	1762 (26.4)	3541 (28.4)	5303 (27.7)
Obese (≥30)	1958 (29.4)	3593 (28.9)	5551 (29.0)
Missing (%)	43.3	33.5	37.3
Ever smoker	2448 (34.6)	4464 (34.8)	6912 (34.7)
Missing (%)	39.9	31.4	34.7
Pre‐existing hypertension	1004 (8.5)	1771 (9.5)	2775 (9.1)
Patient IMD 2015			
Quintile 1 (least deprived)	2256 (19.2)	4171 (22.3)	6427 (21.1)
Quintile 2	2273 (19.4)	4053 (21.7)	6326 (20.8)
Quintile 3	2312 (19.7)	3766 (20.1)	6078 (20.0)
Quintile 4	2323 (19.8)	3559 (19.0)	5882 (19.3)
Quintile 5 (most deprived)	2572 (21.9)	3159 (16.9)	5731 (18.8)
Missing (%)	0.3	0.1	0.1
Obstetric and birth characteristics			
Primiparity	3730 (56.6)	5940 (60.3)	9670 (58.9)
Missing (%)	44.0	47.4	46.1
Multiple birth	242 (2.9)	298 (2.4)	540 (2.6)
Missing (%)	29.6	33.7	32.1
Induction of labour	3542 (44.7)	5393 (45.2)	8935 (45.0)
Missing (%)	32.6	36.2	34.8
Caesarean birth	4431 (37.7)	6603 (35.3)	11 034 (36.2)
Year of birth			
2000–2004	3707 (31.5)	5114 (27.3)	8821 (28.9)
2005–2009	3740 (31.8)	6750 (36.1)	10 490 (34.4)
2010–2014	3276 (27.8)	5475 (29.3)	8751 (28.7)
2015–2018	1043 (8.9)	1378 (7.4)	2421 (7.9)
HDP characteristics			
Type of hypertensive disease of pregnancy			
Gestational hypertension	7920 (67.3)	13 122 (70.1)	21 042 (69.0)
Preeclampsia	3846 (32.7)	5595 (29.9)	9441 (31.0)
Hypertensive complications^a^			
Eclampsia	197 (1.7)	300 (1.6)	497 (1.6)
Placenta separation	94 (0.8)	111 (0.6)	205 (0.7)
HELLP syndrome	79 (0.7)	73 (0.4)	152 (0.5)
Infant characteristics			
Gestational age at birth (weeks), mean (SD)	38.4 (3.1)	38.8 (2.6)	38.6 (2.8)
Preterm birth (<37 weeks)	1942 (16.5)	2347 (12.5)	4289 (14.1)
Low birthweight (<2500 g)	1272 (15.5)	1535 (12.5)	2807 (13.7)
Missing (%)	30.4	34.2	32.7

*Note*: Column percentages presented.

Abbreviations: SWC, six week check; BMI, body mass index; IMD, index of multiple deprivation; HDP, hypertensive disorders of pregnancy; HELLP, Hemolysis, Elevated Liver enzymes and Low Platelets; SD, standard deviation.

^a^ Statistics not presented for pulmonary oedema because of small numbers.

### 3.2 SWC and subsequent adverse hypertension‐related care and outcomes

Overall, 56.4% of women had a BP measurement recorded in primary care notes in the first 4–12 weeks postpartum; this was higher among women who had a SWC compared to women who did not have a SWC (66.2% vs. 40.9%, see also Figure 2 for temporal trend). Only 44.5% of women had a BP measurement recorded after 12 weeks postpartum and up to 1 year postpartum; again, this was more common in those who had had a SWC (47.4% vs. 39.8%, Table 2). However, there was little evidence of a difference by SWC status in recorded hypertension, or in prescribing of antihypertensive medication after 12 weeks (Table 2). Recorded incident CVD events up to 1 year postpartum were higher in women who attended a SWC, but absolute numbers were small (343 (1.8%) vs. 168 (1.4%)).

**TABLE 2:** Short‐term outcomes (1 year postpartum) among women with and without a six‐week check‐up (SWC).

	Did not have a SWC	Had a SWC	Total
	*n* = 11 766	*n* = 18 717	*N* = 30 483
	*n* (%)	*n* (%)	*n* (%)
Incident CVD 1 year postpartum	168 (1.4)	343 (1.8)	511 (1.7)
BP status post‐SWC & 1 year postpartum^a^			
BP measure (No)	7081 (60.2)	9845 (52.6)	16 926 (55.5)
BP measure (Yes)	4685 (39.8)	8872 (47.4)	13 557 (44.5)
Normal	3036 (64.8)	5906 (66.6)	8942 (66.0)
Abnormal	1649 (35.2)	2966 (33.4)	4615 (34.0)
Anti‐hypertensive medication use post‐SWC & 1 year postpartum	373 (3.2)	684 (3.7)	1057 (3.5)
Perineal or pelvic pain	128 (1.1)	244 (1.3)	372 (1.2)
Urinary or fecal incontinence	68 (0.6)	151 (0.8)	219 (0.7)
Sexual dysfunction	129 (1.1)	277 (1.5)	406 (1.3)
Postpartum depression	1305 (11.1)	2551 (13.6)	3856 (12.6)

Abbreviations: BP, blood pressure; SWC, six week check; CVD, cardiovascular disease.

^a^ Any measure post‐SWC and up to 1 year postpartum.

### 3.3 SWC and short‐term postpartum‐specific outcomes

Recording of postpartum conditions such as urinary/fecal incontinence, pelvic or perineal pain, or sexual dysfunction was rare, and there was little evidence that this differed by SWC status (Table 2). However, postpartum depression was identified in 12.6% of pregnancies and was more commonly recorded in those who had had a SWC (overall 13.6 vs. 11.1%) (Table 2). Table 3 shows timing of depression diagnosis in those who did and did not have a SWC—almost 3/4th of the diagnosis was made within six months postpartum.

**TABLE 3:** Depression by time of diagnosis (during 1 year postpartum) among women with and without a six‐week check‐up (SWC).

	Did not have a SWC	Had a SWC	Total
	*n* = 11 766	*n* = 18 717	*N* = 30 483
	*n* (%)	*n* (%)	*n* (%)
**Cumulative prevalence**			
0–3 months postpartum	573 (4.9)	1111 (5.9)	1684 (5.5)
0–6 months postpartum	924 (7.9)	1792 (9.6)	2716 (8.9)
0–9 months postpartum	1155 (9.8)	2280 (12.2)	3435 (11.3)
0–12 months postpartum	1305 (11.1)	2551 (13.6)	3856 (12.6)
**Period prevalence of first diagnosis**			
0–3 months postpartum	573 (4.9)	1111 (5.9)	1684 (5.5)
3–6 months postpartum	351 (3.0)	681 (3.6)	1032 (3.4)
6–9 months postpartum	231 (2.0)	488 (2.6)	719 (2.3)
9–12 months postpartum	150 (1.3)	271 (1.4)	421 (1.4)

Abbreviation: SWC, six week check.

## 4 DISCUSSION

This study of 30 483 women who experienced HDP in a pregnancy that resulted in a birth between 2000 and 2018, showed approximately 3 in 5 women received a maternal postpartum GP check at 6–8 weeks but there were disparities in the provision or uptake. Women who were younger, from more deprived areas, who experienced more severe disease, pregnancy complications, or a preterm or caesarean birth all less likely to have a SWC recorded. BP measurements after a SWC and up until one‐year postpartum were recorded in 56% of women, and were more common in women who had a SWC. The results suggest that only a quarter of women with a record of ongoing hypertension were receiving anti‐hypertensive medications. However, CVD‐related outcomes were relatively rare and no evidence for a difference was observed by SWC status. Postpartum depression up to 12 months postpartum was seen in 12.6% of women and was more commonly observed in women who had had a SWC. However, recording of other common postpartum conditions such as incontinence or pelvic/perineal pain was very low, and these conditions did not differ much between women who had and did not have an SWC.

There are very few studies that describe the prevalence of the SWC in women with HDP. Estimates from the same data source (CPRD), using the same code list to identify SWC, report higher coverage among the general population: in births from 2008 to 2014 coverage was reported as 73.5%,^12^ while the prevalence in the equivalent period in our study was 60.7%, falling to 55.0%. By 2015–2018, coverage in the general population had fallen to 62%^10^ but a greater reduction was seen in our study population where equivalent figures are between 58.1% and 56.8%. It is recommended that after HDP, women have a BP check at 6 weeks and this may be in a hospital setting or within a specialist service.^6^ In these circumstances, they may not also make a SWC appointment at the GP. A survey of 260 women who had preeclampsia in 2001–02 found 99% self‐reported a postnatal check and of these 75% were at the GP,^13^ which may partially explain the lower prevalence we observed.

Our finding that women with severe HDP are less likely to have a GP SWC is surprising but also important. A possible explanation is that if a woman has severe HDP then they are seen by a specialist or in hospital. However, the hospital checks may not follow the advised content of a GP SWC, and therefore we do not know if the same general postpartum care is delivered. A qualitative study of clinicians caring for postnatal women who had HDP showed that lack of postnatal care plans, uncertainty regarding responsibility for care, and women's lack of awareness about the importance of postnatal follow‐up are important reasons for women with HDP to not have a SWC.^14^ BP measurements were not recorded in the first 12 weeks for 44% of women in our study. Furthermore, among women who did have the SWC, just 47.4% had a BP measurement, which was a recommended part of the SWC for all women during the study period and particularly important for those who had recently experienced HDP. A survey in 2000–01 found 93% of women who had experienced preeclampsia reported that their BP was measured at their SWC with the GP.^13^ Our findings for 2000–01 indicate that between 45% and 54% have BP recorded, which highlights poor recording of BP in electronic health records which is necessary for ongoing monitoring and care.

In our population of women with HDP, there was little difference in the proportion of women who had a diagnosis of hypertension, prescription of antihypertensive medication or incident CVD in the first year postpartum among those who did or did not have a SWC. Detection or recording of common non‐HDP related postpartum conditions was low and again, little difference was observed by SWC. Given the remit of the SWC this is concerning, and may reflect the unstructured nature of many checks during the study period, where the onus was on women to raise their concerns.^10^ Notably, self‐reported surveys find far higher prevalence of postpartum conditions such as urinary and fecal incontinence. A study of 653 women with HDP^15^ found self‐reported urinary incontinence in 28.6% of respondents, while we identified relevant codes in just 0.7%. It is not possible to know if the low prevalence of postpartum outcomes is due to the doctors not raising it during the SWC, women not disclosing or seeking help, or inaccurate coding.

Common mental health problems in the postpartum period are seen in about 15%–20% of new mothers, with depression and anxiety accounting for the majority of ill health.^16^, ^17^ The previously mentioned study reported postpartum depression in 11.7% of women at 3 months postpartum,^15^ where we observed 9% at the same time point and 13% overall. However, that study used a screening questionnaire while we identified depression from routine records, which may partially explain this difference. We found that postpartum depression was more commonly recorded in the women who have had a SWC. It is acknowledged that postpartum depression is poorly recognized and treated in primary care, and that a lack of willingness to seek help and concern about the response from medical professionals are recognized barriers to seeking help for perinatal mental health problems.^18^, ^19^ It is plausible that the SWC facilitates a conversation about mental health, builds rapport and empowers women to seek support when they need it. However, this positive effect would only occur in practices where time and care is taken over the SWC, an appointment that is considered rushed and unsatisfactory by many women.^20^

Recommended postpartum check‐ups for women are not unique to the UK. The latest guidelines by the World Health Organization are to have at least three postnatal contacts during the first 6 weeks postpartum in addition to a minimum of 24—hour stay after birth.^21^ The American College of Obstetricians and Gynecologists recommend postpartum evaluation of women by within first 3 weeks after birth, and a follow‐up, if required, and a comprehensive examination by 12 weeks' postpartum.^22^ A study that utilized 2009–20 211 data from the Pregnancy Risk Assessment and Surveillance System (PRAMS) reported that women who were younger, not married, multiparous, had a preterm birth and had low education, and no insurance (possible indicators of deprivation) were more likely to miss their postnatal visit at 4–6 weeks.^23^ These results are similar to the findings from our study.

We used both primary care and hospital admissions data to identify pregnancies with HDP in women included in CPRD, ensuring a large sample of women with known HDP.^24^ The CPRD GOLD data are broadly representative of the UK population in terms of age and ethnicity.^25^ However, our sample may not be representative of all practices in the UK in terms of geography and size.^7^ We used previously validated electronic health record‐algorithms (https://www.caliberresearch.org/portal/) or code lists (see Table S1) or developed comprehensive lists with clinical input, as needed, to derive key variables. However, it remains possible that code lists fail to identify or misclassify some consultations or diagnoses. Electronic health records data is only as good as the coding, and it is not possible to tell how much the prevalence of the postpartum outcomes was affected by poor coding. For BP data, it is not clear if measurements taken in other settings (eg. home, health visitor, hospital) and reported to the GP are entered into the software in a way that would allow us to see the figures. We were unable to account for health‐seeking behaviors in this population. Informed presence bias in data from electronic health records such as the CPRD describes a situation where consultations, diagnosis or treatment for one condition may make it more likely to be diagnosed with another.^26^ However, given that all women in this study had a recent diagnosis of HDP, this potential bias is minimized. Another limitation is bias resulting from exclusion of women (*n* = 19 021) who were not registered with a GP for at least a year post‐birth. These women were excluded to ensure good quality and more complete data on the short‐term outcomes for the first year postpartum. However, these women who moved from the GP practice for various reasons may be different from the women included in the study leading to potential bias.

## 5 CONCLUSION

This study highlights the low prevalence of SWC records and disparities in SWC provision for poorer, younger women, and those who had a more difficult pregnancy or birth, even within a group of women with HDP—where they have all previously been identified as in need of additional postpartum care. Monitoring and adequate recording of BP across this period should be prioritized to reduce the potential impact on longer‐term maternal morbidity or mortality. There is the potential for the care of high‐risk women, such as those who experienced HDP, to focus only on a specific morbidity and overlook their broader postpartum health. Clearer guidance for GPs regarding the content and conduct of the SWC is now in place,^27^ and should allow more standardized and equitable care to be provided. Following the introduction of the SWC as an essential service under the GP contract in 2020,^5^ and the continuing emphasis on postpartum health in the Women's Health Strategy,^5^ the SWC offers an important opportunity to improve care for all women. It is important that women with HDP receive a general SWC including BP measurement either with their GP, or ensure their GP is fully informed, so that they too can benefit from improved postpartum services.

## AUTHOR CONTRIBUTIONS

Claire Carson conceptualized and supervised the project. Rema Ramakrishnan and Diane Korb developed the analysis plan with input from Claire Carson and Yangmei Li. Diane Korb and Rema Ramakrishnan cleaned, prepared and managed the data and conducted the statistical analysis with input from Marian Knight and Claire Carson. Rema Ramakrishnan and Claire Carson drafted the article with input from all the other authors. All authors contributed to interpretation of the findings, revised the manuscript critically for important intellectual content and approved the final version.

## FUNDING INFORMATION

MK is an NIHR Senior Investigator (grant ref. NIHR303806). This research is part‐funded by the National Institute for Health Research (NIHR) Policy Research Programme, conducted through the NIHR Policy Research Unit in Maternal and Neonatal Health and Care, PR‐PRU‐1217‐21202. The views expressed are those of the authors and not necessarily those of the NIHR or the Department of Health and Social Care.

## CONFLICT OF INTEREST STATEMENT

The author have nothing to report.

## ETHICS STATEMENT

The current study is part of a larger project exploring the impact of abnormal BP values after HDP on incident CVD, and has been approved by the CPRD Research Data Governance process on January 24, 2022 (Protocol number: 21_000725).

## Supporting information


Table S1.
Table S2.
Table S3.

## Data Availability

Data may be obtained from a third party and are not publicly available. The data that support the findings of this study are available from Clinical Practice Research Datalink (CPRD). Restrictions apply to the availability of these data, which were used under licence for this study. The data were provided by the CPRD under a contractual agreement that does not permit the sharing of data.
