# Supplementary material for: Maternal postpartum six‐week check and short‐term health outcomes for women with hypertensive disorders in pregnancy: An observational study using the Clinical Practice Research Datalink (CPRD)
Source: Acta Obstet Gynecol Scand. 2025 Feb 25;104(5):937–47. doi: 10.1111/aogs.15068 (PMC11981094; doi:10.1111/aogs.15068)
Supplement: Supplementary file 1 — Table S1. Table S2. Table S3. [file AOGS-104-937-s001.docx]

**SUPPLEMENTARY MATERIAL**

**Does the maternal postpartum six-week check in primary care improve short-term health outcomes for women with hypertensive disorders in pregnancy?**

Rema Ramakrishnan, Diane Korb, Yangmei Li, Marian Knight, Claire Carson4

This section describes code lists that were used to define antihypertensive medication use, perineal or pelvic pain, urinary or faecal incontinence, sexual dysfunction, and postpartum depression. Antihypertensive medication use was based on product codes listed at: <https://data.bris.ac.uk/datasets/k38ghxkcub622603i5wq6bwag/prod_antihypertensives.v1.txt>

The variables for perineal or pelvic pain, urinary or faecal incontinence, and sexual dysfunction were derived based on the medcodes listed in Tables S1, S2, and S3, respectively. The presence of depression postpartum was based either on a diagnosis of depression or symptoms/history of depression along with usage of antidepressant medications.^1^

**Table S1**. Code list used to define perineal/pelvic pain

| **CPRD Medcode** | **Description** |
| --- | --- |
| 2781 | C/O pelvic pain |
| 16547 | Other pelvic pain ‐ female |
| 4706 | Bony pelvic pain |
| 9920 | [D]Pelvic and perineal pain |
| 9811 | [D] Pelvic pain |
| 29400 | C/O perineal pain |
| 7248 | [D] Perineal pain |

**Table S2**. Code list used to define urinary and faecal incontinence

| **CPRD Medcode** | **Description** |
| --- | --- |
| 583 | Urgency of micturition |
| 1929 | Stress incontinence |
| 2739 | Incontinence care |
| 3182 | Stress incontinence |
| 3283 | [D] Incontinence of urine |
| 3887 | Urge incontinence of urine |
| 4375 | Enuresis NOS |
| 5196 | Double incontinence |
| 5705 | Dribbling of urine |
| 5844 | Stress incontinence – symptom |
| 6161 | Incontinence of urine |
| 7649 | [D]Micturition syncope |
| 8028 | [D] Urgency of micturition |
| 9020 | Bladder training |
| 12138 | Continence care |
| 13421 | Bladder: incontinent |
| 13422 | Bladder: occasional accident |
| 13423 | Bladder- continence assessment |
| 13424 | Bladder-incontinence assessment |
| 13428 | Bowels - continence |
| 15400 | [D] Incontinence of urine – NOS |
| 15918 | H/O: stress incontinence |
| 17320 | [D] Urge incontinence |
| 17620 | Stress incontinence – female |
| 17637 | Incontinence control |
| 20728 | Pad test for incontinence |
| 22095 | Seen by continence nurse |
| 25899 | Referral to incontinence clinic |
| 25901 | Referral to continence nurse |
| 29039 | Health education - continence |
| 29040 | Promotion of continence |
| 29192 | Referral to continence nurse |
| 30981 | Urinary bladder control |
| 31220 | [D] Urethral sphincter incontinence |
| 40789 | Continence assessment |
| 43234 | [V] Admission for bladder training |
| 43931 | Discharge by continence nurse |
| 45492 | Under care of continence nurse |
| 45495 | Provision of incontinence appliance |
| 46614 | Procedures to aid continence |
| 47963 | Urinary bladder control NOS |
| 48601 | Incontinence control |
| 49417 | Continence reassessment |
| 52763 | [X] Other specified urinary incontinence |
| 73228 | [X]Other difficulties with micturition |
| 93952 | Urge to pass urine again shortly after finishing voiding |
| 94021 | Insertion retropubic dev fem stress urinary incontinence NEC |
| 94673 | Seen by continence nurse |
| 98767 | Insertion retropubic device stress urinary incontinence NEC |
| 100658 | International consultn incontinence questionnaire short form |
| 100822 | OS other operations on the anal sphincter control continence |
| 101647 | OPCS continence disability scale |
| 104801 | OS other operations on anal sphincter control continence NOS |
| 106548 | Continence care equipment available at home |
| 107787 | Incontinence-associated dermatitis |
| 109624 | Functional urinary and faecal incontinence |
| 110001 | Functional urinary incontinence |
| 1437 | [D] Incontinence of faeces |
| 3381 | Incontinent of faeces |
| 5196 | Double incontinence |
| 6083 | Incontinent of faeces symptom |
| 6364 | Chronic constipation with overflow |
| 13426 | Bowels: incontinent |
| 15555 | [D] Incontinence of faeces NOS |
| 27623 | [D] Sphincter ani incontinence |
| 31256 | Bowels-incontinence assessment |
| 43222 | Bowels incontinence assessment |
| 68374 | Other operations on the anal sphincter to control continence |

**Table S3**. Code list used to define sexual dysfunction

| **CPRD Medcode** | **Description** |
| --- | --- |
| 94316 | Sexual intercourse difficult |
| 15264 | H/O: dyspareunia |
| 37089 | [X]Psychogenic dyspareunia |
| 2708 | Dyspareunia due to non- psychogenic cause in the female |
| 365 | Dyspareunia |
| 21253 | Psychogenic dyspareunia |
| 21089 | [X]Nonorganic dyspareunia |
| 27693 | sexual function problem |
| 6362 | lack of libido |
| 20109 | psychogenic vaginismus |
| 34336 | [x]inhibited orgasm |
| 48890 | [x]psychosexual development disorder, unspecified" |
| 31196 | advice for sexual dysfunction |
| 29694 | h/o: sexual dysfunction problem |
| 23534 | unspecified psychosexual dysfunction |
| 9485 | [x]sex dysfunction not caused by organic disorder or disease |
| 10550 | [x]psychogenic anorgasmia |
| 2708 | dyspareunia due to non-psychogenic cause in the female |
| 89011 | derogatis sexual functioning inventory |
| 10811 | h/o: sexual problem - female |
| 2259 | inhibited sexual desire |
| 28283 | [x]lack or loss of sexual desire |
| 19745 | [x]nonorganic vaginismus |
| 15766 | vaginismus due to non-psychogenic cause |
| 89012 | dsfi - derogatis sexual functioning inventory |
| 2172 | vaginismus |
| 809 | frigidity |
| 56603 | [x]hypoactive sexual desire disorder |
| 30442 | [x]psychogenic vaginismus |
| 28571 | sexual dysfunction counselling |
| 16060 | [v]problem with sexual function |
| 94316 | sexual intercourse difficult |
| 19082 | inhibited female orgasm |
| 21122 | [x] lack of libido |
| 37089 | [x]psychogenic dyspareunia |
| 25851 | counselling for sexual dysfunction |
| 20620 | [v]counselling related to sexual attitude |
| 58681 | menopause: sexual advice |
| 20133 | psychosexual dysfunction nos |
| 42056 | [x]sexual aversion and lack of sexual enjoyment |
| 44683 | "[x]oth sex dysfunction, not caused by organic disorder/disease" |
| 71712 | sex therapy technique |
| 17073 | [v]other sex counselling |
| 1526 | psychosexual counselling |
| 40257 | other psychosexual disorders |
| 24483 | [x]anhedonia sexual |
| 48953 | [x]unspec sex dysfunction not caused by organic disorder/dis |
| 48285 | psychosexual therapy |
| 61908 | "[x]sex counselling,unspecified" |
| 13680 | referral to psychosexual clinic |
| 27344 | other psychosexual disorder nos |
| 60716 | [x]female sexual arousal disorder |
| 27791 | [x]sexual relationship disorder |
| 71972 | generic psychosexual therapy |
| 15649 | psychosexual dysfunction |
| 1525 | psychosexual disorder nos |
| 18332 | [x]orgasmic dysfunction |
| 30414 | [x]other psychosexual development disorders |
| 71968 | relationship orientated psychosexual therapy |

**Reference**

1. Tianyi FL, Li Y, Alderdice F, et al. The association between conception history and subsequent postpartum depression and/or anxiety: Evidence from the Clinical Practice Research Datalink 1991-2013. *J Affect Disord* 2022(1573-2517)
